# Reorganization of Istituti Fisioterapici Ospitalieri, an oncological and dermatological clinical and research center, to face the coronavirus health emergency: adopted measures and metrics of success to achieve and keep a COVID-19-free status

**DOI:** 10.1186/s13046-020-01675-y

**Published:** 2020-09-01

**Authors:** Assunta De Luca, Francesco Ripa di Meana, Branka Vujovic, Aldo Morrone, Chiara Degirolamo, Gennaro Ciliberto, Tiziana Lavalle

**Affiliations:** 1 Quality Assessment and Clinical Risk Manager Unit, Istituti Fisioterapici Ospitalieri (IFO), Rome, Italy; 2 Istituti Fisioterapici Ospitalieri (IFO), Rome, Italy; 3 IRCCS San Gallicano Dermatological Institute, Istituti Fisioterapici Ospitalieri (IFO), Rome, Italy; 4 Florence, Italy; 5 IRCCS Istituto Nazionale Tumori Regina Elena, Istituti Fisioterapici Ospitalieri (IFO), Rome, Italy; 6 Organizational and Human Capital Development Unit, Istituti Fisioterapici Ospitalieri (IFO), Rome, Italy

**Keywords:** Cancer care, COVID-19, Telemedicine, Triage, Surveillance

## Abstract

**Background:**

A pronounced polarization of healthcare resources and workforce towards the prevention of the rapid spread of SARS-CoV-2 occurred at the expenses of the majority of chronic diseases and cancer, thus jeopardizing continuity of care and therapy outcomes.

**Main body of the abstract:**

In this challenging and overwhelming scenario, our Institute confirmed its mission to provide expert cancer care. Here, we provide a report of strategic decisions made and of articulated measures developed to limit virus spreading while striving to make our hospital closer to patients.

**Conclusions:**

We hope our experience may serve as a resource to inform clinical care models in case of future epidemiological outbreaks.

## Background

The Istituti Fisioterapici Ospitalieri (IFO) comprise two scientific institutes: the Regina Elena National Cancer Institute (IRE) & the Dermatological Institute S. Gallicano (ISG), both located in Rome, Lazio, Italy. Despite the overwhelming challenges the entire healthcare community faced due to coronavirus disease 2019 (COVID-19) diffusion in our Region, IFO confirmed its mission to provide expert care without compromising continuity of assistance for its patients. Here we describe the strategic decisions made and the plans developed to limit the spread of COVID-19, while taking the opportunities going along with the outbreak. We also report on the effects of our actions, hoping that our experience could be helpful in promoting resilience, emergency plan and continuity of care provision within a public healthcare system.

### Activation of a crisis unit and adoption of general measures

Since the beginning of the COVID-19 outbreak in Italy, we designed institutional protocols and internal guidelines early on to guarantee cancer treatment for our patients while minimizing COVID-19-related risks ensuring a safe environment for care providers. The first measure was the activation of a Crisis Unit, chaired by the Medical Director and including the main representatives of different units involved in the virus containment^1^, that consisted in meeting once a week to discuss the current clinical scenario, and updating institutional protocols and internal guidelines through a shared decision-making process. The weekly meeting of the Crisis Unit was organized more frequently when needed until 30 May 2020 when institutional activities were fully restored. Afterwards, the meetings, held via a web-based conference platform, were attended every 14 days.

Protective and organizational measures have been implemented to provide healthcare professionals with the necessary training needed against the spread of the virus, including prevention, containment measures and hygiene recommendations: use of personal protective equipment (PPE), wearing PPE equipments in healthcare settings, PPE disposal in biohazard containers, use of screening questionnaires for patients admitted to hospital. The training of healthcare providers and PPE supply had a key role.

Further measures to preserve safety of our employees have been also implemented, including the use of smart working via a dedicated platform developed upon the healthcare emergency. Until 18 May 2020, about 250 employees were currently on smart working. Residential meetings were replaced by video conferences.

Our Institute adopted internal and external communication measures to highlight the health risks and behavior necessary to manage those risks. The Press Office and Communication Office worked synergistically to inform and constantly update both healthcare providers and patients regarding the virus prevention and containment measures by using printed brochures, visual alerts and poster distributed in the hospital.

### Filters to and within the hospital

Restrictions of patients and employees’ access to the hospital, employ assistance and cancer center continuity of care support were implemented.

#### Patient admission to the hospital

**Fig. 1:**
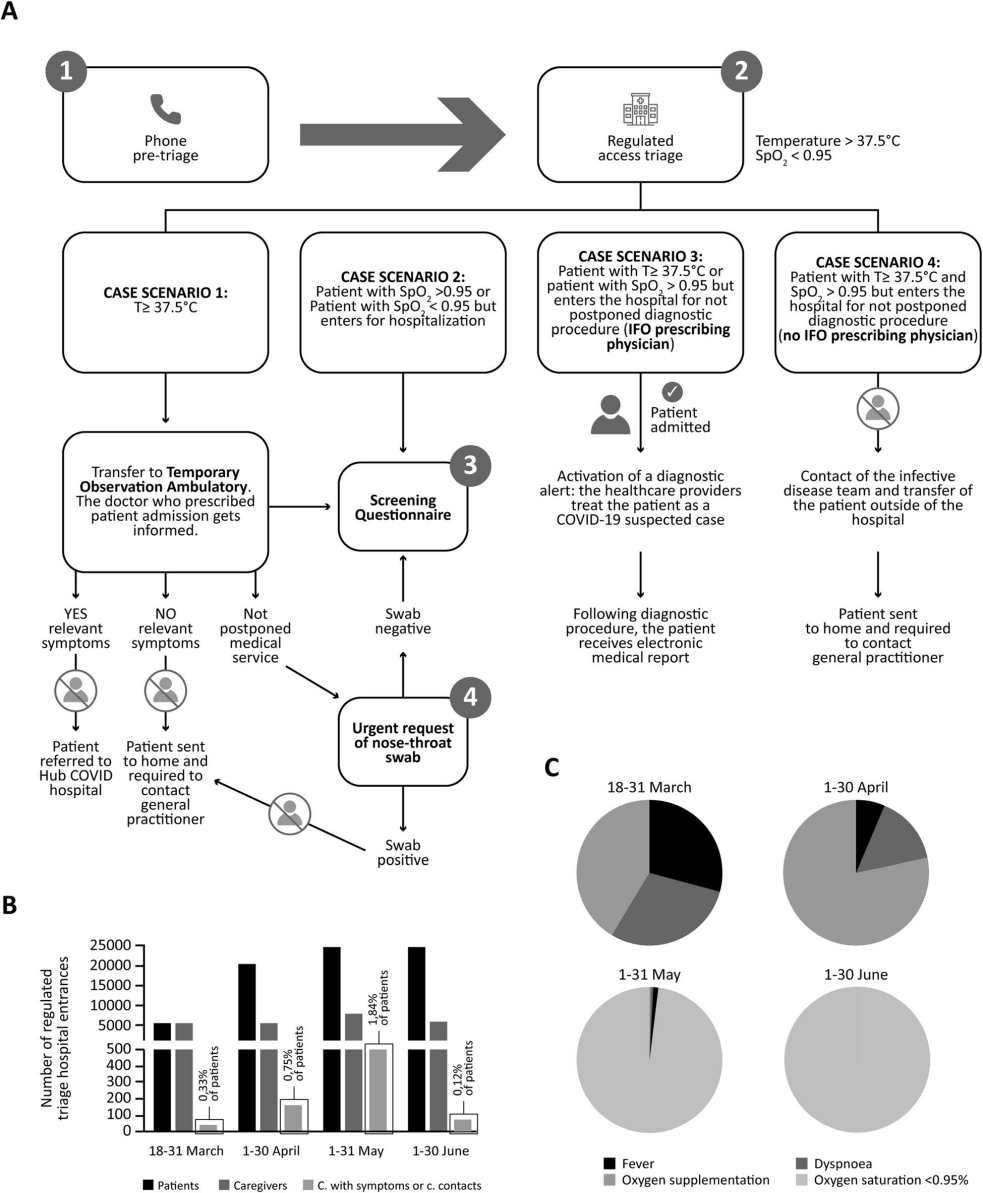
Flowchart of patients admitted to the hospital (**a**), number of hospital admissions during lockdown (**b**) and number of cases with symptoms or with critical contacts (**c**)

A triage was applied before entering the hospital. Patients were receiving a phone call 24–48 h before hospital admission to monitor their health status and to potentially identify suspected cases. If tested positive, patient was not admitted to the hospital. Furthermore, no visitors were allowed to IFO for the duration of this crisis. Figure 1a shows a flowchart pertaining to patients admitted to the hospital and encompassing four filters. The prerequisite for triage was the presence of acute symptoms, defined as fever (> 37.5 °C), assessed by a thermal scanner, suspicion of respiratory infection, and at least one of the following data: respiratory rate > 30/min, oxygen saturation (SpO_2_) < 0.95% without oxygen supplementation, and dyspnea. The patient was also interviewed about potential contact with confirmed or suspected COVID-19 patients. The triage allowed us to detect an increasing proportion of suspected patients over time, ranging from 0.3–1.2%, up to June 2020, who were either referred to hospitals dedicated to COVID-19 treatment or sent home, thus avoiding their admission to the wards whenever possible (Fig. 1b). As of 30 June 2020, we identified 756 suspected patients. Restriction measures dropped visitors and/or caregivers percentage proportion from 42.9% in March 2020 to 9,15% in June 2020. Over time, the parameter most frequently associated with the condition of “symptomatic case” and/or a “case with critical and/or risk contacts” changed from being in contact with a COVID-19-confirmed case and showing dyspnoea during the early stages of the outbreak to the presence of oxygen saturation < 0.95% (Fig. 1c).

**Fig. 2:**
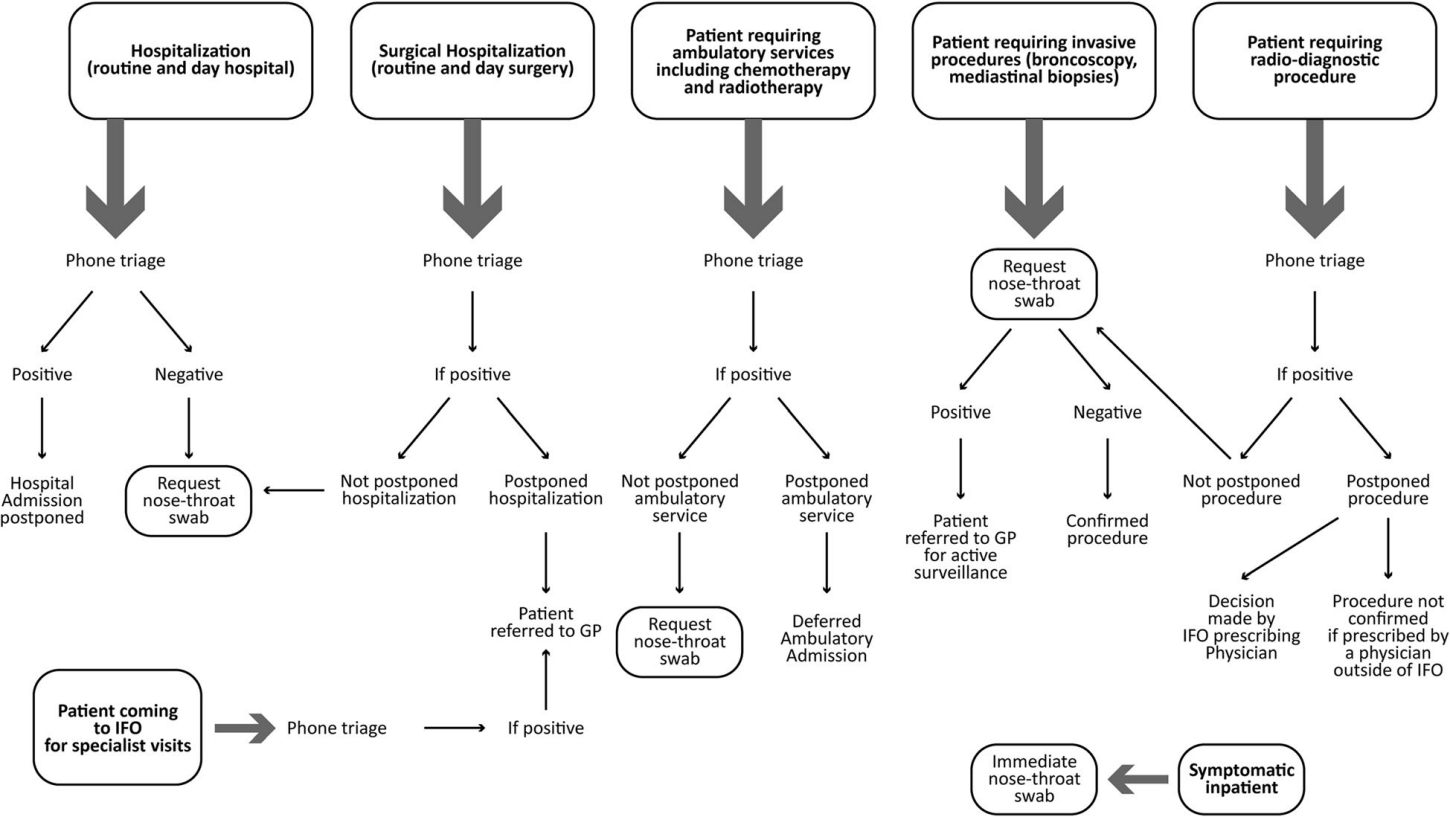
Prescribing criteria to request nose–throat swabs

The nose–throat swab is the current standard procedure to confirm a COVID-19 diagnosis. Relying on a Microbiology and Virus Unit and associated laboratories, our Institute has been part of the Lazio CoroNET network and has analyzed almost 21,576 nose–throat swabs (from 30 March to 30 June 2020). Eighty-seven nose-throat swabs have been analyzed for an external company located in our Region. To optimize the access to swabs while preserving health system sustainability and limiting unjustifiable requests, we have defined prescribing criteria for nose–throat swabs by identifying seven potential case scenarios as illustrated in Fig. 2.

#### Active surveillance and epidemiological survey of healthcare workers

**Fig. 3:**
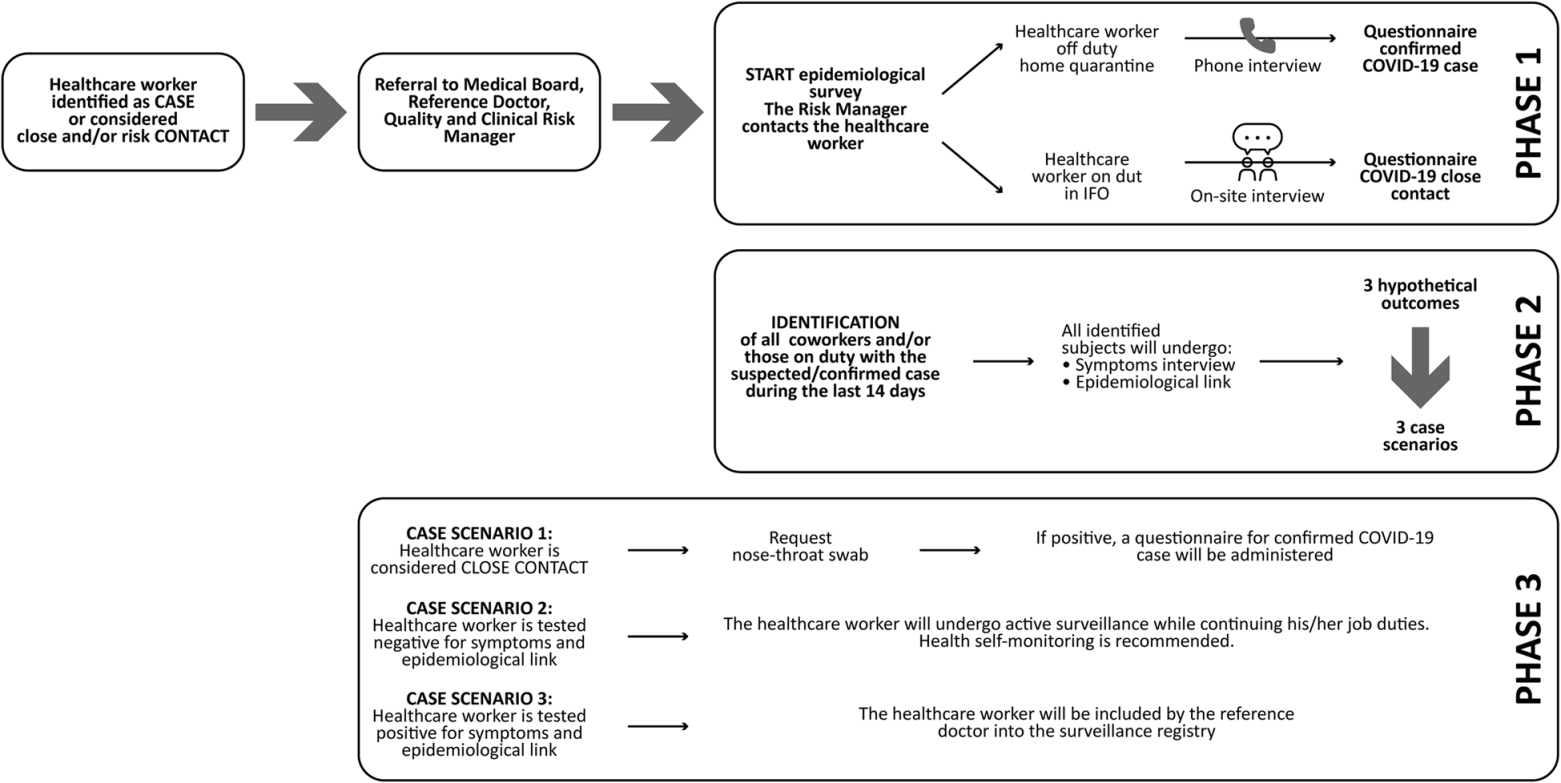
Flowchart pertaining the actions taken within IFO to manage contagion containment and isolation of suspected cases

Healthcare personnel taking care of patients with cancer need to be safeguarded thus following PPE guidelines. A surveillance registry has been established to identify those, who may be categorized as contacts at risk and subsequently monitored by the hospital risk manager via a risk assessment questionnaire. As a result, healthcare workers can be categorized as low and high risk. Low-risk healthcare workers undergo active surveillance for 2 weeks monitoring their body temperature and symptoms daily. High-risk healthcare workers are tested for COVID-19 by nose–throat swab and if they test positive, they are quarantined at home; if they test negative, they undergo active surveillance for 2 weeks. Figure 3 shows a flowchart pertaining the actions taken within IFO to manage contagion containment and isolation of suspected cases.

A total of 370 subjects underwent surveillance and 2,151 were screened between 18 March and 30 June 2020. Reverse transcription polymerase chain reaction (RT-PCR) was positive in 8 healthcare workers and in 16 patients (seven inpatients and nine outpatients). A total of 1,598 serological tests have been carried out of which 1,563 for employees and 35 for homeless people. Overall, 51 employees were found positive and underwent nose-throat swabs with only one being positive and subsequently undergoing active surveillance.

### Reorganization of care provision to make our hospital closer to patients

Our institution reshaped the provision of care to address the urgency of not deferrable procedures (either surgeries, biopsy or chemotherapy/radiotherapy) while banning the access only for follow-up visits, according a case-by-case evaluation of the risk/benefit ratio [1].

To reduce potential viral exposure at pharmacy departments and avoid in-person hospital visits to refill prescriptions, at home delivery service has been quickly implemented for those receiving either oral anticancer therapy or biologics. During the national lockdown, we have reached 119 patients with about 450 delivered treatments, thus markedly reducing hospital visits while promoting better patients’ quality of life and favoring improved adherence to therapy.

Patients who are not currently receiving active therapy may be well-suited for telemedicine consultation and/or routine follow-up. A switch to telemedicine has been recently recommended in breast cancer patients [2] and virtual visits have been proposed as promising tools to ensure continuity of cancer services and assistance to patients [3]. We have rapidly developed a dedicated telemedicine platform, named *#IFOConTeOnline*, through expedited physician credentialing, training and modification based on changing regulations. With 9,178 web interactions on our platform, we have reached more than 2,000 patients and provided telemedicine services, consultations, symptom control and counselling to both cancer patients and their caregivers as well as to external patients with mild and ordinary dermatological conditions [4].

To support people living with cancer during COVID-19 isolation and to mitigate the effects of quarantine on mental and physical well-being, we relied on digital technology to implement two phone helplines to promote patient mental health and coping with the pandemic crisis.

Finally, we have realized that ensuring psychological support of frontline workers is also necessary to prevent burnout and attrition.

## Conclusions

The COVID-19 outbreak has presented both challenges and learning opportunities for cancer centers [5]. As suggested by Trapani et al., the key to success in COVID-19 and cancer is to ensure a continuum of healthcare never disconnecting the cancer cause from the population health needs [6].

Overall, the proactive management and containment measures, including the triage protocols, have significantly aided the identification of as many as 756 patients with suspected symptoms related to COVID-19, thus limiting their admission to our cancer center. Furthermore, the activation of the active surveillance registry and the epidemiological survey allowed us to closely monitor 370 employees, to test over 200 of them and guarantee to those tested positive the immediate treatment and evaluation in referral hospitals for COVID-19. A total of 1,598 serological tests have been carried out of which 1,563 for employees and 35 for homeless people. Overall, 51 employees were found positive and underwent nose-throat swabs with only one being positive and subsequently undergoing active surveillance. We have reshaped our provision of care by preserving elective and day surgeries and urgent ambulatory services and deferring only routine follow-up visits. Nevertheless, we acknowledge that, in the long term, the capacity to undertake diagnostics and elective surgery should be further expanded to address a growing backlog of delayed diagnosis and unmet needs.

## Data Availability

All data in our study are available upon request.

## Abbreviations

IFO: Istituti Fisioterapici Ospitalieri
IRE: Istitute Regina Elena
ISG: Istitute San Galllicano
PPE: Personal protective equipment
SARS-CoV-2: Severe acute respiratory syndrome coronavirus 2
SpO2: Oxygen saturation

## Footnotes

1 Medical Director, who was the chairman, Pharmacy Unit Director, Economic Resources Director, Medical Dept Unit Director, Health Professional Unit Director, Human Resources Director, Quality Assessment and Clinical Risk Manager, Nurse Reference for prevention of care-related infections, Responsible of the Project “Welcome in hospital”, Responsible of the Diagnostic Team, Education Reference Unit, Directors of three Clinical and Research Departments.
